# That Resolution

**Published:** 1896-07

**Authors:** 


THAT RESOLUTION.—Shades of our departed great, hide your diminished heads. What would the lamented, the great CUPPLES (peace to his ashes and all honor to his memory) say to the proposition to put four homeopaths and two eclectics on a board of medical examiners with six members of the beloved State Association—to the welfare and honor of which he devoted his life? What would he say could he be here and read that puerile

"Resolution?" He would hang his head in shame, and blush for the departed greatness of the Texas State Medical Association. The "Resolution"—we reproduce it,—says:

"WHEREAS, The Texas State Medical Association adopted the report of the committee wherein it was recommended that the homeopaths and eclectics be recognized in the law [bill] in order to get a constitutional law passed, governing the practice of medicine, and as some uninformed persons may construe this into an endorsement of the homeopaths and eclectics;

"Therefore, we positively declare that we do not recognize them only so far as they are recognized by statutes and constitution of the State of Texas to enable us to have a medical law passed in the State, but that we stand by the Code of the American Medical Association, and will expel any doctor of the State of the Texas Medical Association who will lower the dignity of regular medicine as to meet them in consultation."

Of all the ambiguous, senseless, stupid productions—attempts to justify a mistake—the above is the worst we have ever seen. It reflects no credit upon the author, and certainly none on the Association. In the first place, the recognition by the statutes or the constitution of the State is complete, unqualified. In the law the homeopaths and eclectics and all other paths are recognized as "schools of medicine," an absurdity on its face, for there *are no* "schools of medicine," as Dr. Brittain, the author of the resolution, and every member knows full well. To so recognize them ourselves is to tacitly admit that we—the regular medical profession, are the other "school"—an admission that involves for us acceptance of the name "allopaths," and puts us on an even and exact footing with all other "pathies!" The writer repudiates it, and will not accept it. * * *

Again, some "uninformed persons may construe this into an endorsement of the homeopaths and eclectics." We do not see how even the best informed persons can escape the conclusion that it is a "recognition;" (no one has ever accused the Association of "endorsing" the homeopaths and eclectics) and indeed, it is declared in the next breath that "we *do* recognize them"—"to enable us to have a medical law!" Here is an affirmation and a denial in the same breath. "We declare we do not recognize them." "We recognize them"—"to enable us to have a medical law,"—"so far as they are recognized by statutes and constitution,"—which is entire, *complete* recognition as "schools of medicine." * * *

But the climax of absurdity is reached when the resolution affirms that we "stand by the Code of the American Medical

Association"—after having committed a gross breach of the spirit if not the letter of that Code. It is like "standing by" a man—after spitting in his face. * * * And in the fullness of his wrath—the Association's wrath—we "will expel any doctor" * * * "who will lower the dignity of regular medicine as to meet them [homeopaths and eclectics] in consultation." This, after "lowering the dignity of regular medicine" to the last notch; lowered it to the extent of asking to be put in affiliation with the *other two schools*, (?) both of which we denounce as quacks. Could stultification be more complete?

---

THE CONSTITUTION OF THE STATE, it is claimed, says "no preference shall ever be shown by law to the several schools of medicine." This was clearly intended to apply to the distribution of patronage, and has reference to the appointments to the various government positions, the officers of the State institutions, etc. I do not see how, in the making of a law at the request of the medical profession, defining the qualifications of those entrusted with the privilege of practicing medicine, the objection could possibly be wrung in that a preference is made for any "schools." Yet the State Association, in framing this bill which asks for a board composed of representatives of the "three schools," seems to anticipate such objection on the part of the legislature; it suggests it, really, when, if left alone, and simply asked for such a proper bill, it perhaps would never have occurred to them. Suppose the State Medical Association should send in a bill to define the qualifications of practitioners of medicine, and state that by "practice of medicine" is meant only what is understood by laymen as the "regular profession," and by some as "allopathy"; that it is sought only to fix a standard of qualifications for those who essay the practice of regular medicine, and has no reference whatever to the practice of any "sect" or "pathy," it is hard to conceive wherein any "preference would be involved in the granting of such request. The proposition of the State Association to have a mixed board is entirely gratuitous and unnecessary. It is a most unfortunate mistake, and we sincerely hope it may be reconsidered before the matter goes to the legislature.

